# Treatment of Varicose Veins

**Published:** 1843-11

**Authors:** 


*Treatment of Varicose Veins.*—Moulinie recommends the following method of operating for the cure of varicose veins, as not being attended with the bad results that not unfrequently succeed to other similar operations:

"A fold of the skin is taken up in a direction parallel to that of the vein, and at some distance above the varicose part, an incision is then made across this fold down to the cellular tissue around the vein, disturbing the part by dissection as little as possible. An eye-probe, armed with a silk or fine thread, is then passed under the vein, and the latter is then tied. An incision is often made across the vein, a little above the ligature, for the purpose of preventing any inflammation of the vein spreading by continuity along it. The skin is then brought together by strapping, in order to protect the vein from the contact of air as completely as possible."—*Amer. Journ. of the Med. Sci.*, from *Prov. Med. Journ.*, July, 22, 1843, from *Bonheur en Chirurgie, &c.*

